# Multiagent cooperation and competition with deep reinforcement learning

**DOI:** 10.1371/journal.pone.0172395

**Published:** 2017-04-05

**Authors:** Ardi Tampuu, Tambet Matiisen, Dorian Kodelja, Ilya Kuzovkin, Kristjan Korjus, Juhan Aru, Jaan Aru, Raul Vicente

**Affiliations:** 1 Computational Neuroscience Lab, Institute of Computer Science, University of Tartu, Tartu, Estonia; 2 Department of Mathematics, ETH Zürich, Zürich, Switzerland; Tianjin University of Technology, CHINA

## Abstract

Evolution of cooperation and competition can appear when multiple adaptive agents share a biological, social, or technological niche. In the present work we study how cooperation and competition emerge between autonomous agents that learn by reinforcement while using only their raw visual input as the state representation. In particular, we extend the Deep Q-Learning framework to multiagent environments to investigate the interaction between two learning agents in the well-known video game Pong. By manipulating the classical rewarding scheme of Pong we show how competitive and collaborative behaviors emerge. We also describe the progression from competitive to collaborative behavior when the incentive to cooperate is increased. Finally we show how learning by playing against another adaptive agent, instead of against a hard-wired algorithm, results in more robust strategies. The present work shows that Deep Q-Networks can become a useful tool for studying decentralized learning of multiagent systems coping with high-dimensional environments.

## Introduction

In the ever-changing world biological and engineered agents need to cope with unpredictability. By learning from trial-and-error an animal, or a robot, can adapt its behavior in a novel or changing environment. This is the main intuition behind reinforcement learning [1, 2]. A reinforcement learning agent modifies its behavior based on the rewards it collects while interacting with the environment. By trying to maximize these rewards during the interaction an agent can learn to implement complex long-term strategies.

When two or more agents share an environment the problem of reinforcement learning is notoriously more complex. Indeed, most of game theory problems deal with multiple agents taking decisions to maximize their individual returns in a static environment [3]. Collective animal behavior [4] and distributed control systems are important examples of multiple autonomous actors in dynamic environments. Phenomena such as cooperation, communication, and competition may emerge in reinforced multiagent systems.

While the distributed nature of learning in multiagent systems offers benefits (e.g., inherent parallelism, scalability, or robustness versus failure of some of the agents), new challenges such as how to define good learning goals arise. Also there are few guarantees about the convergence and consistency of learning algorithms [3, 5, 6]. This is so because in the multiagent case the environment state transitions and rewards are affected by the joint action of all the agents. Thus, the value of an agent’s action depends also on the actions of the others and hence each agent must keep track of each of the other learning agents, possibly resulting in an ever-moving target [3, 5, 6]. In general, learning in the presence of other agents requires a delicate trade-off between the stability and adaptive behavior of each agent [3, 5, 6].

Due to the astronomic number of possible states in any realistic environment until recently algorithms implementing reinforcement learning were either limited to simple settings or needed to be assisted by additional information about the dynamics of the environment [7]. Recently, however, the Swiss AI Lab IDSIA [8] and Google DeepMind [7, 9] have produced spectacular results in applying reinforcement learning to very high-dimensional and complex environments such as video games. In particular, [7, 9] demonstrated that AI agents can achieve superhuman performance in a diverse range of Atari video games. Remarkably, the learning agent only uses raw sensory input (screen images) and the reward signal (increase in game score). The proposed methodology, the so called Deep Q-Network (DQN), combines a convolutional neural network for learning feature representations with the Q-learning algorithm [10]. The fact that the same algorithm was used for learning very different games might suggest it has potential for more general purpose applications [7, 11].

The present article builds on the work of [7]. Instead of a single agent playing against a hardcoded algorithm, we explore how multiple agents controlled by autonomous DQNs learn to cooperate and compete while sharing a high-dimensional environment and being fed only raw visual input. This is an extension to the existing multiagent reinforcement learning studies done in simple grid worlds or with agents already equipped with abstract high-level sensory perception [3, 12, 13]. In particular, using the video game *Pong* and manipulating the rewarding schemes we describe the agents’ emergent behavior with a set of behavioral metrics. We show that the agents develop successful strategies for both competition and cooperation, depending on the incentives provided by rewarding schemes. We also tune the rewarding schemes in order to study the intermediate states in the progression from competitive to collaborative behavior. Finally, we illustrate how learning by playing against another learning agent results in more robust strategies than those achieved by a single agent trained against a stationary hard-wired algorithm. Agents trained in multiplayer mode perform very well against novel opponents, whereas agents trained against a stationary algorithm fail to generalize their strategies to novel adversaries.

## Materials and methods

### Q-learning algorithm

The goal of reinforcement learning is to find the policy *π*—a set of rules to select an action in each possible state—that would maximize the agent’s accumulated long term reward in a dynamical environment. The problem is especially challenging when the agent must learn without explicit information about the dynamics of the environment or the rewards.

The reinforcement learning problem is usually modeled as a Markov decision process (MDP) giving rise to a sequence of observed, states, actions and rewards—*s*_0_, *a*_0_, *r*_1_, *s*_1_, *a*_1_, *r*_2_, *s*_2_, …, *s*_*T*_. The *s*_*t*_ is the state of the environment, *a*_*t*_ the action taken by the agent and *r*_*t*_ the reward received by the agent at time-step *t*. One episode (game) lasts T time steps, and different episodes can be of different length. The goal of the agent is to maximize the sum of rewards *r*_1_ + *r*_2_ + … + *r*_*T*_ (the total game score).

One popular method to solve MDPs is Q-learning [14]. Q-learning is based on estimating the expected total discounted future rewards (the quality) of each state-action pair under a policy *π*:

$$Q_{\pi }\left(s_{t},\;a_{t}\right)\;=\;E\;\left[r_{t+1}\;+\;\gamma r_{t+2}\;+\;\gamma^{2}r_{t+2}\;+\;\ldots \;+\;\gamma^{T-t}r_{T}\;|\;\pi \right].$$ (1)

Here *γ* is a discount rate between 0 and 1 that makes future rewards less valuable than immediate ones and helps to cope with infinite MDPs. The optimal quality value is then Q*(*s*_*t*_, *a*_*t*_) = max_*π*_ *Q*_*π*_(*s*_*t*_, *a*_*t*_). Hence an optimal policy is easily derived from the optimal values by selecting the highest valued action in each state, and the problem only amounts to obtaining accurate Q-values.

Given state *s*, action *a*, reward *r* and next state *s*′, it is possible to approximate Q*(*s*, *a*) by iteratively solving the Bellman recurrence equation [1]:

$$Q_{i+1}\left(s,\;a\right)\;=\;E\;\left[r\;+\;\gamma \;\max_{a^{\prime }}Q_{i}\left(s^{\prime },\;a^{\prime }\right)\right].$$ (2)

When the state-action space is small enough for the Q-values to be represented as a lookup table, this iterative approximation is proved to converge to the true Q-values [15], provided that all state-action pairs are regularly sampled. However, the combinatorial explosion of the number of possible states in even a modest-size environment makes this table based implementation of Q-learning unfeasible. This problem can be partially overcome by function approximation methods. Instead of storing each Q-value, their aim is to learn a function that maps state-action pairs to their respective Q-values.

### Deep Q-learning algorithm

Deep Q-network (DQN) builds on standard Q-learning by approximating the Q-function using a non-linear neural network. The neural network, parametrized by *θ*, is trained to minimize the loss function:

$$L\left(\theta \right)\;=\;E\;[{\left(\underset{\text{target}}{\underset{︸}{r\;+\;\gamma \;\max_{a^{\prime }}Q\left(s^{\prime },\;a^{\prime };\;\theta^{\prime }\right)}}-\underset{\text{prediction}}{\underset{︸}{Q(s,\;a;\;\theta )}}\right)}^{2}]$$ (3)

Notice that the formula closely reassembles the iterative update rule of the Bellmann equation mentioned above (Eq 2). Essentially, the goal is to minimize the difference between the current estimation of the Q-value (prediction), and an updated estimate (target) that combines the obtained reward and an estimation of the quality of the next state.

There is no proof of convergence for Q-learning with non-linear function approximators. To overcome learning instability, all experiences (*s*, *a*, *r*, *s*′) are stored in a “replay memory” and are sampled uniformly as training examples. This ensures that the examples are uncorrelated and do not drive the policy to a local minima. Furthermore, a separate target network (with parameters *θ*′ in the formula above) is used for estimating the maximal Q-value. The target network’s weights are updated at certain intervals to be equal with those of the main network. Between updates these target Q-values remain unchanged and provide some much needed stability.

To balance the exploitation of the current best known Q-values with the exploration of even better options, DQN uses a simple *ϵ*-greedy policy that samples a random action with probability *ϵ* (instead of always picking the action with maximal Q-value). When *ϵ* is annealed over time, this simple strategy is known to work just as well as more complex exploration strategies [1].

There exist several possible adaptations of the Q-learning algorithm for the multiagent case [5]. However, this is an open research area and theoretical guarantees for multiagent model-free reinforcement learning algorithms are scarce and restricted to specific types of tasks [3, 5]. In practice the simplest method consists of using an autonomous Q-learning algorithm for each agent in the environment, thereby using the environment as the sole source of interaction between agents. In this work we use this method due to its simplicity, decentralized nature, computational speed, and ability to produce consistent results for the range of tasks we report. Therefore, in our tests each agent is controlled by an independent DQN with architecture and parameters as reported in [7].

### Adaptation of the code for the multiplayer paradigm

We needed to introduce several adaptations to the original code published with [7] to allow training multiple agents simultaneously. These changes are summarized in supplementary information (SI) section.

### Game selection

We chose to use the *Pong* game environment in our study for reasons summarized in the SI. In short, there are three advantages for illustrating our results with *Pong*: i) the *Pong* game has a real-time two player mode, ii) DQNs are good in *Pong* and iii) the game is well-known and can be easily understood by the reader.

In *Pong* each agent corresponds to one of the paddles situated on the left and right side of the screen (see screenshots in Results section). There are 4 actions that each of the two agents can take: move up, move down, stand still, and fire (to relaunch the ball or to start the game). Which action is taken is decided by the corresponding DQN for both agents separately.

### Rewarding schemes

A central aim of this work is to study the emergence of different types of collective behavior depending on how the agents are rewarded.

We adjust the rewarding scheme by simply changing the reward *ρ* a player receives when putting the ball past the opponent (when scoring). This essentially means, that we change the values on the main diagonal of the payoff matrix, given in Table 1. The reward for conceding is kept fixed at -1. This way we are able create several different games within the same *Pong* environment. Examples of the used rewarding schemes are given in the following subsection*s.

**Table 1:** Rewarding schemes to explore the transition from competitive to the cooperative strategy.

	L player scores	R player scores
L player reward	*ρ*	−1
R player reward	−1	*ρ*

For the cases we study *ρ* ∈ [−1, 1]. Example: with *ρ* = −0.5, when the left player scores, it receives −0.5 points and the right player receives -1 points.

#### Score more than the opponent (fully competitive)

In the traditional rewarding scheme of Pong, also used in [7], the player who scores a point gets a positive reward of size 1 (*ρ* = 1). The player conceding a point is penalized with a reward of -1. This makes it essentially a zero-sum game, where a positive reward for the left player implies a negative reward of the same size for the right player and vice versa. Notice that *ρ* = 1 is the only case where the rewards of the two players sum up to zero, for all *ρ* < 1 the sum of rewards is negative.

#### Loosing the ball penalizes both players (fully cooperative)

In this setting we want the agents to learn to keep the ball in the game for as long as possible. To achieve this, we penalize both of the players whenever the ball goes out of play—both scoring and conceding lead to a punitive reward of −1 (*ρ* = −1). Which of the players lets the ball pass does not matter and no positive rewards are given.

#### Transition between cooperation and competition

The above two rewarding schemes define fully competitive and fully collaborative tasks. To study the behavioral patterns lying between these two extremes we gradually reduce the reward difference between scoring and conceding. The reward for conceding is kept fixed at -1, while a set of intermediate values are given to the *ρ* parameter.

### Training procedure

In all of the experiments we let the agents learn for 50 epochs, 250000 time steps each. We limit the learning to 50 epochs because the Q-values predicted by the network have stabilized (see S1 Fig). Due to using a frame skipping technique the agents see and select actions only on every 4th frame [7]. In this article we always talk about the frames the agents actually see and so we use “visible frame”, “frame” and “time step” interchangeably.

During the training time, as in [7], the exploration rate (proportion of actions chosen randomly) decreases from an initial 1.0 to 0.05 in the first million time steps and stays fixed at that value. A more detailed description of the training procedure and parameters can be found in [7].

After each epoch snapshots of the DQNs are stored to facilitate the future study of the training process. We also track the average maximal Q-value, which has been previously used as an indicator of training convergence (see SI).

To guarantee a fair comparison between different rewarding schemes, we need the training signal to be equally strong in all cases. Rewards are the signal that agents use to evaluate their performance and that they learn from. For each scheme, we multiply the rewards with a normalization coefficient so that the sum of their absolute values would be equal. The rewarding schemes and the normalization coefficients are listed in S1 Table.

### Collecting the game statistics

To obtain quantitative measures of the agents’ behavior in the *Pong* environment, we identified and counted specific events in the game, e.g. bouncing of the ball against the paddle or the wall. We used Stella [16] integrated debugger to detect the memory locations containing information about these events. Based on these counts we defined a set of behavioral metrics listed below.

The statistics are collected after each training epoch by letting the DQNs (in their current state) play against each other for 10 games, each game initialized with a different random seed (In *Pong* one game consists of multiple exchanges and lasts until one of the agents reaches 21 points.). During this testing phase the exploration rate is set to 0.01. The behavioral measures we used are the following:

- **Average *paddle-bounces* per point** counts how many times the ball bounces between two players before one of them scores a point. Randomly playing agents almost never hit the ball. Well trained agents hit the ball multiple times in an exchange. Hereafter we refer to this statistic as *paddle-bounces*.
- **Average *wall-bounces* per paddle-bounce** quantifies how many times the ball bounces from top and bottom walls before it reaches the other player. It is possible to hit the ball in an acute angle so that it bounces the walls several times before reaching the other player. Depending on the strategy, players might prefer to send the ball directly to the other player or use the wall bounces. Hereafter we refer to this statistic as *wall-bounces*.
- **Average *serving time* per point** shows how long it takes for the players to restart the game after the ball was lost (measured in frames). To restart the game, the agent who just scored has to send a specific command (fire). Depending on the rewarding scheme the agents might want to avoid restarting the game. Hereafter we refer to this statistic as *serving time*.

## Results

### Emergence of competitive agents

**Fig 1:**
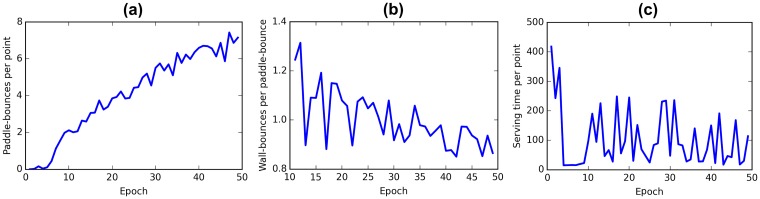
Evolution of the behavior of the competitive agents during training. (a) The number of paddle-bounces increases indicating that the players get better at catching the ball. (b) The frequency of the ball hitting the upper and lower walls decreases slowly with training. The first 10 epochs are omitted from the plot as very few paddle-bounces were made by the agents and the metric was very noisy. (c) Serving time decreases abruptly in early stages of training- the agents learn to put the ball back into play. Serving time is measured in frames.

In the full competitive (zero-sum) rewarding scheme each agent obtains an immediate reward when the ball gets past the other agent and an immediate punishment when it misses the ball. Initially the agents fail to hit the ball at all but with training both agents become more and more proficient. The learning of both agents progresses continuously. Fig 1 summarizes the evolution of the quantitative descriptors of behavior during training.

Qualitatively we can report that by the end of training both agents play the game reasonably well. First of all, both players are capable of winning regardless of who was the one to serve. Secondly, the exchanges can last for a considerable amount of paddle-bounces (Fig 1a) even after the speed of the ball has increased. Thirdly we observe that the agents have learned to put the ball back in play rapidly (Fig 1c).

**Fig 2:**
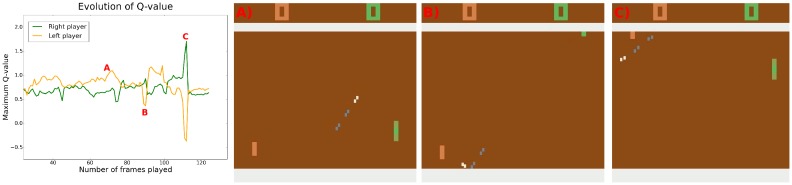
A competitive game—game situations and the Q-values predicted by the agents. A) The left player predicts that the right player will not reach the ball as it is rapidly moving upwards. B) A change in the direction of the ball causes the left player’s reward expectation to drop. C) Players understand that the ball will inevitably go out of the play. See supporting information for videos illustrating other game situations and the corresponding agents’ Q-values.

Fig 2 illustrates how the agents’ predictions of their rewards evolve during an exchange. A first observation is that the Q-values predicted by the agents are optimistic, both players predicting positive future rewards in most situations. The figure also demonstrates that the agents’ reward expectations correlate well with game situations. More precisely, one can observe that whenever the ball is travelling towards a player its reward expectation drops and the opponent’s expectation increases. This drop occurs because even a well trained agent might miss the ball (at least 5% of the actions are taken randomly during training), resulting in −1 and +1 rewards for the two players respectively.

We also note that the Q-values correlate with the speed of the ball. The faster the ball travels, the bigger are the ups and downs in Q-values—possibly because there is less time to correct for a bad initial position or a random move.

Interestingly, one can also notice that as soon as the serving player has relaunched the ball its reward expectation increases slightly. This immediate increase in Q-value makes the agents choose to serve the ball as soon as possible and thus explains the decrease in serving time (Fig 1c).

See supporting information for a short example video displaying the behavior of the agents in different game situations and the corresponding Q-values.

### Emergence of collaborative agents

In the fully cooperative rewarding scheme both agents receive an immediate punishment whenever the ball get past either of them. Thus, the agents are motivated to keep the ball alive. The agents get no positive rewards and the best they can achieve is to minimize the number of times the ball is lost.

**Fig 3:**
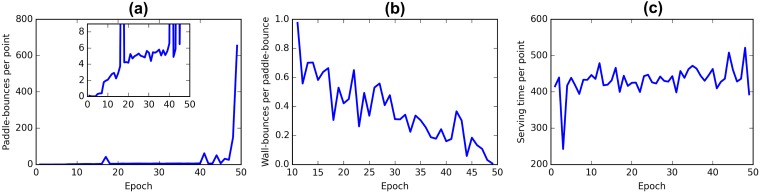
Evolution of the behavior of the collaborative agents during training. (a) The number of paddle-bounces increases as the players get better at reaching the ball. (b) The frequency of the ball hitting the upper and lower walls decreases significantly with training. The first 10 epochs are omitted from the plot as very few paddle-bounces were made by the agents and the metric was very noisy. (c) Serving takes a long time—the agents learn to postpone putting the ball into play.

The evolution of the quantitative descriptors of behavior during cooperative training is shown on Fig 3. The emergent strategy after 50 epochs of training can be characterized by three observations: (i) the agents have learned to keep the ball for a long time (Fig 3a); (ii) the agents take a long time to serve the ball (Fig 3c) because playing can only result in negative rewards; and (iii) the agents prefer to pass the ball horizontally across the field without touching the walls (Fig 3b).

On Figs 1b and 3b we see that the number of wall bounces decreases with training in both cases. High values in early stages of training are an artifact of the game—the ball is in many cases launched at an acute angle and hits the wall once before reaching a player. In early stages players make only a few touches and the wall-bounce per paddle-bounce ratio is artificially high. With training agents keep the ball alive longer and this effect is diluted. Notice that in the case of competitive strategy the wall-bounces ratio remains near 1 even after 50 epochs of training (Fig 1b), whereas in cooperative mode this measure gradually tends to zero (Fig 1b). This happens because cooperative agents gradually learn to pass the ball horizontally across the field. It seems that hitting the ball at acute angles is a characteristic of competitive behaviour.

**Fig 4:**
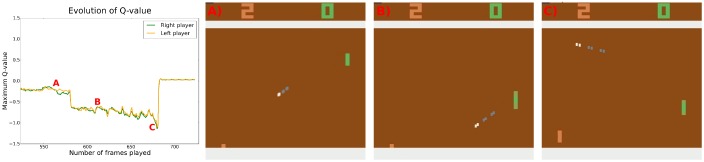
Cooperative game—game situations and the Q-values predicted by the agents. A) The ball is moving slowly and the future reward expectation is not very low—the agents do not expect to miss the slow balls. B) The ball is moving faster and the reward expectation is much more negative—the agents expect to miss the ball in the near future. C) The ball is inevitably going out of play. Both agents’ reward expectations drop accordingly. See supporting information for videos illustrating other game situations and the corresponding agents’ Q-values.

On Fig 4 an exchange between collaborative agents is illustrated. Just like the competitive agents, the collaborative agents learn that the speed of the ball is an important predictor of future rewards, faster balls increase the risk of mistakes. The clear drop in the predicted Q-values in situation B compared to situation A is caused by the ball travelling faster in situation B.

In the exchange illustrated on Fig 4 the agents eventually miss the ball. In some exchanges, however, the players apply a coordinated strategy where both agents place themselves at the upper border of the playing field and bounce the ball between themselves horizontally (see S2 Fig). Whenever a random action takes them away from the upper edge, they move back towards the edge in the next time step. Being at the edge of the field minimizes the effect of random actions—random movements to only one of two directions are possible. Arriving to this stable situation happens in every game, but not necessarily in every exchange.

See supporting information for a video illustrating the evolution of the agents’ learning progression towards the final converged behavior and the coordinated strategy.

### Progression from competition to cooperation

Besides the two cases described above, we also ran a series of simulations with intermediate rewarding schemes. Our aim here is to describe the emergent behaviors when the immediate reward received for scoring a point (*ρ*) is decreased.

**Fig 5:**
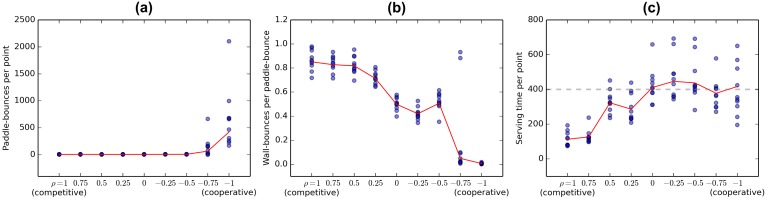
Progression of behavioral statistics when passing from competitive to collaborative rewarding scheme. Each blue dot corresponds to the average of one game. Red line depicts the average across games (also given in S2 Table). (a) The game lasts longer when the agents have a strong incentive to collaborate. (b) Forcing the agents to collaborate decreases the proportion of angled shots that bounce off the walls before reaching the opposite player. Notice the two aberrant values for *ρ* = −0.75 correspond to games where the agents never reach the collaborative strategy of keeping the ball alive by passing it horizontally. (c) Serving time decreases when agents receive stronger positive rewards for scoring.

On Fig 5, the quantitative behavioral metrics are plotted for decreasing values of *ρ* in order to give a better overview of the trends. S2 Table summarises these results numerically. The statistics are collected after agents have been trained for 50 epochs and are averaged over 10 game runs with different random seeds.

The average number of paddle-bounces the two agents make in an exchange (Fig 5a, S2 Table) increases abruptly at *ρ* = 0.75, because the agents sometimes manage to reach the collaborative strategy described in section 2.2 and illustrated on S2 Fig. Compared to fully collaborative mode, the *ρ* = 0.75 agents reach this strategy less frequently.

The number of wall-bounces per paddle-bounce stays high for *ρ* > 0 and seems to have a downwards trend when we decrease the reward from scoring from 0 to -1. Above we suggested that hitting the ball at an acute angle might be a characteristic of aggressive play. In Discussion, we argue that the observed flat-and-downwards trend agrees with optimal strategies for these intermediate rewarding schemes.

The average time the agents take to relaunch a game is summarized on Fig 5c. Notice that when the agent never chooses to relaunch a game, it takes on average 400 timesteps to be restarted by the random actions. We see that for all *ρ* ≤ 0 the agents indeed always avoid relaunching the ball. For *ρ* > 0 the average serving time increases with decreasing rewards for scoring. In the Discussion we argue that the observed trends agree with the optimal policies for these *ρ* values.

### Comparison of agents trained in multiplayer and single-player modes

Training against another adaptive agent is often referred to as self-play and has been associated with benefits such as evolving more general strategies [17, 18]. Typically agents are first trained in a supervised manner, learning from examples. Self-play is then used in a second phase of learning to enhance the performance even further [19]. To demonstrate the benefits of self-play for DQNs with no supervised pre-training, we now proceed to evaluate our competitive agents’ capacity to play against new opponents.

**Fig 6:**
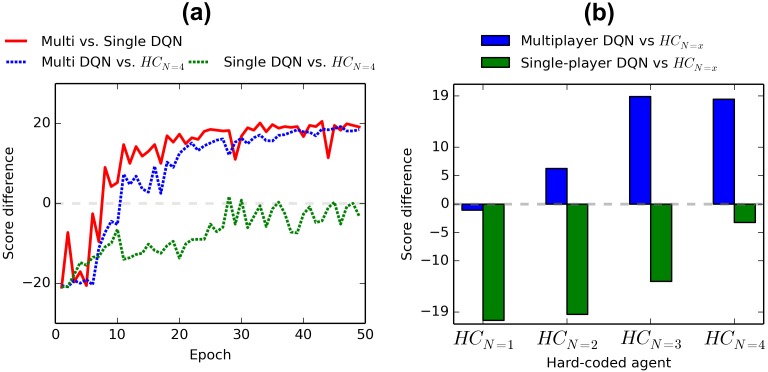
Results of games between multiplayer DQN, single-player DQN and four hand-coded algorithms. The values correspond to an average of 10 games with different random seeds. Score difference means the points scored by the agent mentioned first minus the points of the agent mentioned second. (a) Multi and Single DQN’s performance against each other and against *HC*_*N*=4_ in function of training time. (b) Scores of Single DQN and Multi DQN agents against 4 versions of a handcoded agent trying to keep the center of the paddle level with the ball. N refers to the number of frames a selected action is repeated by the algorithm before selecting a new action (the smaller the better).

First we make our competitive agent trained in multiplayer mode (*multiplayer DQN*) play against a DQN agent trained in single-player mode (*single-player DQN*; trained against the algorithm built into the Pong game, as in [7]). After each training epoch we match the multiplayer DQN against a single-player DQN trained for the same number of epochs. The average score differences (points by multiplayer DQN minus points by single-player DQN) of these games are shown with a red line on Fig 6a. The multiplayer training starts slower than single-player (negative score difference in early epochs), because neither of the players know how to play (in single player mode, the Pong algorithm does). Nevertheless, after 50 epochs the multiplayer DQN defeats the single-player DQN with an average score of 21-2.

Secondly we make both multiplayer DQN and single-player DQN play against 4 variations of a hand-coded algorithm that tries to keep the center of the paddle at the same height with the ball at all times. The algorithm is restricted to select an action only every N frames, with N ranging from 1 (new action every frame) to 4 in the four variations we tested against (*HC*_*N*=1_ .. *HC*_*N*=4_). The chosen action is repeated until the N frames have passed and a new choice is made. When the action selection is more frequent (N is smaller), tracking of the ball becomes smoother and missing the ball less likely. Notice that the DQN agents choose actions every 4 frames (as in [7]).

Fig 6b demonstrates that multiplayer DQN performs better against these new opponents. Single-player DQN loses on average to all the opponents. The multiagent DQN, loses against *HC*_*N*=1_ (the strongest opponent) by only an average of 1.3 points. It defeats *HC*_*N*=2_ by 4.3 points on average and beats *HC*_*N*=3_ and *HC*_*N*=4_ by a score of 21-2. The blue dotted line on Fig 6a shows that multiagent DQN learns to outperform *HC*_*N*=4_ already in early stages of the training process (line crossing above 0), whereas single-player DQN (green dotted line) never outperforms this opponent (nor the others).

In SI we discuss that the single-player DQN has learned to exploit an apparent weakness in the Pong algorithm, leading to a certain type of overfitting and a less generalizable strategy. The combination of this learned strategy being inefficient against other opponents and the single-player agent itself being prone to making simple mistakes (see SI for more details) leads to unsatisfying performance against new adversaries. In multiplayer setting such overfitting is less likely, because both agents are capable of learning from their mistakes.

In all, these results suggest that multiplayer training procedures are less likely to learn very specialized behaviours that are useful only against a specific opponent. Instead, they develop more robust strategies.

## Discussion

Multiagent reinforcement learning has an extensive literature in the emergence of conflict and cooperation between agents sharing an environment [3, 12, 13]. However, most of the reinforcement learning studies have been conducted in either simple grid worlds or with agents already equipped with abstract and high-level sensory perception.

In the present work we demonstrated that agents controlled by autonomous Deep Q-Networks (DQNs) are able to learn a two player video game such as *Pong* from raw sensory data. This result indicates that DQNs might become a useful tool for the decentralized learning of multiagent systems living a high-dimensional environments without the need of manual feature engineering.

In particular, we described how the agents learned and developed different strategies under different rewarding schemes, including full cooperative and full competitive tasks. The emergent behavior of the agents during such schemes was robust and consistent with their tasks. For example, under a cooperative rewarding scheme the two *Pong* agents (paddles) discovered the coordinated strategy of hitting the ball parallel to the *x*-axis, which allowed them to keep the ball bouncing between them for a large amount of time. It is also interesting to note that the serving time, i.e. the time taken by the agent to launch the first ball in a game, was one of the behavioral variables modified during the learning.

We also notice that single-agent training against a stationary hard-wired algorithm exposes the DQN to a limited set of opponent behaviors. In the multiagent setting the strategies of both agents may change considerably during training. This type of training may expose the agents to a more diverse range of opponent behaviors and game situations, thus making them more capable of playing well against an unseen adversary.

### Discussion of optimal strategies for intermediate rewarding schemes

Here, we give a brief discussion on what the optimal strategies would be under different rewarding schemes used in this work. In this theoretical discussion, we consider that both agents are equally skilled and therefore equally likely to win any exchange. In practice we cannot guarantee such equality in all situations, but we do observe that across games the rewards are equally distributed. In the following, we divide this discussion of optimal behaviour to two different phases of the game 1) when the ball is out of play and needs to be served and 2) when the ball is in play.

In the first case an agent needs to decide if it is beneficial for it to relaunch the game. With *ρ* ≤ 0 it is clear that serving is never the optimal choice as any exchange can only lead to negative rewards. In fact, the average expected reward from an exchange is negative for all *ρ* < 1, because the agents are equal and punishments are bigger than rewards. Nevertheless, in case of *ρ* > 0, in specific game situations serving might still be the good choice (e.g. when opponent has placed itself unfavourably are the agent is very likely to score). In general, we would expect the average serving time to increase with decreasing *ρ*. For all non-positive *ρ* we expect the agents to avoid serving in all situations. This is indeed what we observe on Fig 5c.

Let us now consider the case where the ball has already been put into play. Clearly, in the case of *ρ* ≥ 0 an agent should always try to score. Scoring leads to a positive or zero reward and helps avoid a possible negative reward (conceding) in the future. At the other end of the spectrum, with *ρ* = −1, scoring is punished as strictly as conceding and the only strategy for minimizing losses is to keep the ball alive. As described above, this leads to cooperative behaviour.

With −1 < *ρ* < 0, the best possible strategy is still to keep the ball alive forever, but the incentive to discover this strategy is reduced. Remember that both agents are independently trying to maximize their own reward. If the agents are not skillful enough or if the ball is flying too fast, keeping the ball in play for a long time is not probable. In such case, the optimal strategy for an agent might be to compete for the lesser penalty (*ρ* instead of −1), instead of trying to collaborate. By decreasing the reward difference between scoring and conceding we decrease this incentive to compete. We therefore expected the agent to play on average more cooperatively when *ρ* is decreased from -0.25 to -1. In our work we suggest that aggressive play can be estimated by the number of wall-bounces per paddle bounce. This metric (Fig 5b) does indeed stay equally high for *ρ* ≥ 0 and has a decreasing trend when *ρ* is decreased from 0 to -1.

### Limitations

We observe that in the fully competitive (zero-sum) rewarding scheme, the agents overestimate their future discounted rewards. The realistic reward expectation of two equally skillful agents should be around zero, but in most game situations both of our DQNs predict rewards near 0.5 (Fig 2, videos in supporting information). Overestimation of Q-values is a known bias of the Q-learning algorithm and could potentially be remedied by using the novel Double Q-learning algorithm proposed by [20]. Nevertheless notice that biased Q-values do not necessarily mean that the policy would be biased or wrong.

In this work we have used the simplest adaptation of deep Q-learning to the multiagent case, i.e., we let an autonomous DQN to control each agent. In general, we expect that adapting a range of multiagent reinforcement algorithms to make use of DQNs will improve our results and pave the way to new applications of distributed learning in high-dimensional environments.

A larger variety of metrics might have helped us to better describe the behavior of different agents. More descriptive statistics such as average speed of ball and how often the ball is hit with the side of the paddle would have required analyzing the screen images frame by frame. While probably useful quantitative descriptors of behavior, we were limited to the statistics extractable from the game memory. Some of the above-mentioned descriptors were nevertheless used in qualitative descriptions of behaviour.

### Future work

In the present work we have considered two agents interacting in an environment such as *Pong* with different rewarding schemes leading them towards competition or collaboration. Ongoing work is devoted to study the feature representation learning achieved by the different types of agents. In particular, one can make use of guided back-propagation [21] to compare the visual features that activate the hidden nodes of the DQN controllers of competitive and cooperative agents.

Using other games such as *Warlords* we plan to study how a larger number of agents (up to four) organize themselves to compete or cooperate and form alliances to maximize their rewards while using only raw sensory information. It would certainly be interesting to analyse systems with tens or hundreds of agents in such complex environments. This is currently not feasible with the system and algorithms used here.

Convolutional neural networks have become the much needed high-level computational framework against which to contrast data-driven hypotheses of visual processing in the brain [22, 23]. Similarly, we believe that the success (and limitations!) of network architectures endowed with different capabilities [24–26] provide important insights and constraints for how other cognitive processes occur in the brain. A future direction of the present approach is to study of how communication codes [27, 28] and consensus [28–30] can emerge between interacting agents in complex environments without any a priori agreements, rules, or even high-level concepts of themselves and their environment.

## Supporting information

S1 Fig Convergence of deep Q-networks. The convergence of Q-values is a known indicator of the convergence of the learning process of a DQN controlling the behaviour of an agent. Hence, we monitor the *average maximal Q-values* of 500 randomly selected game situations, set aside before training begins. We feed these states to the networks after each training epoch and record the maximal value in the last layer of each of the DQNs. These maximal values correspond to how highly the agent rates its best action in each of the given states and thus estimates the quality of the state itself. On the figure we illustrate the evolution of the Q-value of cooperative, intermediate and competitive agents over the training time.(a) Q-value estimated by the competitive agents, *ρ* = 1. (b) Q-value estimated by the intermediate agents, *ρ* = 0. (c) Q-value estimated by the collaborative agents, *ρ* = −1.
(TIF)

S2 Fig Cooperative strategy allowing to keep the ball indefinitely. By placing themselves at the upper border of the field and bouncing the ball between themselves, the cooperative agents manage to keep the ball in the game indefinitely.
(TIF)

S1 Text Adaptation of the code for the multiplayer paradigm. (PDF)

S2 Text Game selection. (PDF)

S3 Text Comparison of single-player and multiplayer training. (PDF)

S4 Text Access to code. (PDF)

S5 Text Access to videos of gameplay. (PDF)

S1 Table Normalization of rewarding schemes. Rewarding schemes used to explore the behaviours between competitive and the cooperative strategy. Rewards in columns 2 and 3 must be multiplied with the normalization coefficient given in the 4th column to make the learning signal equally strong in all rewarding schemes.
(PDF)

S2 Table Table of behavioral measures for “Progression from competition to collaboration”. Behavioural statistics of the agents as a function of their incentive to score.
(PDF)

S1 File Data underlying the figures. This archive contains data points that are visualized on Figs 1, 3, 5 and 6 and S1 and S2 Tables. Figs 2, 4 and S2 are screenshots and no data associated to them is available.
(ZIP)
