# Supplementary figures and images for: Multiagent cooperation and competition with deep reinforcement learning
Source: PLoS One. 2017 Apr 5;12(4):e0172395. doi: 10.1371/journal.pone.0172395 (PMC5381785; doi:10.1371/journal.pone.0172395)

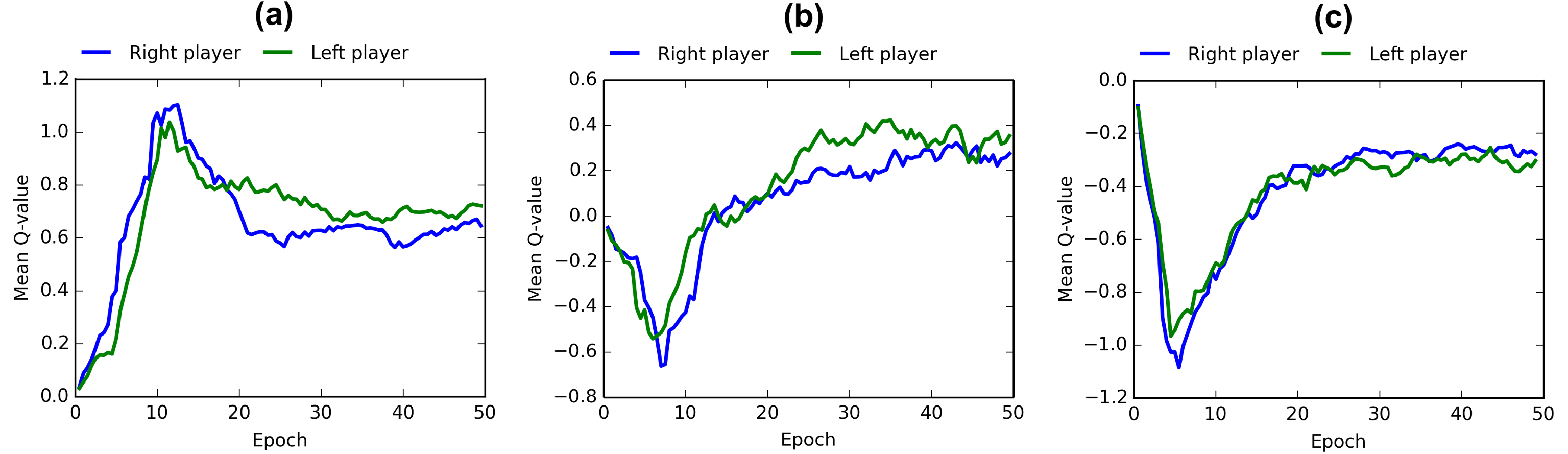

Supplement: S1 Fig — The convergence of Q-values is a known indicator of the convergence of the learning process of a DQN controlling the behaviour of an agent. Hence, we monitor the average maximal Q-values of 500 randomly selected game situations, set aside before training begins. We feed these states to the networks after each training epoch and record the maximal value in the last layer of each of the DQNs. These maximal values correspond to how highly the agent rates its best action in each of the given states and thus estimates the quality of the state itself. On the figure we illustrate the evolution of the Q-value of cooperative, intermediate and competitive agents over the training time.(a) Q-value estimated by the competitive agents, ρ = 1. (b) Q-value estimated by the intermediate agents, ρ = 0. (c) Q-value estimated by the collaborative agents, ρ = −1. (TIF) [file pone.0172395.s001.tif]

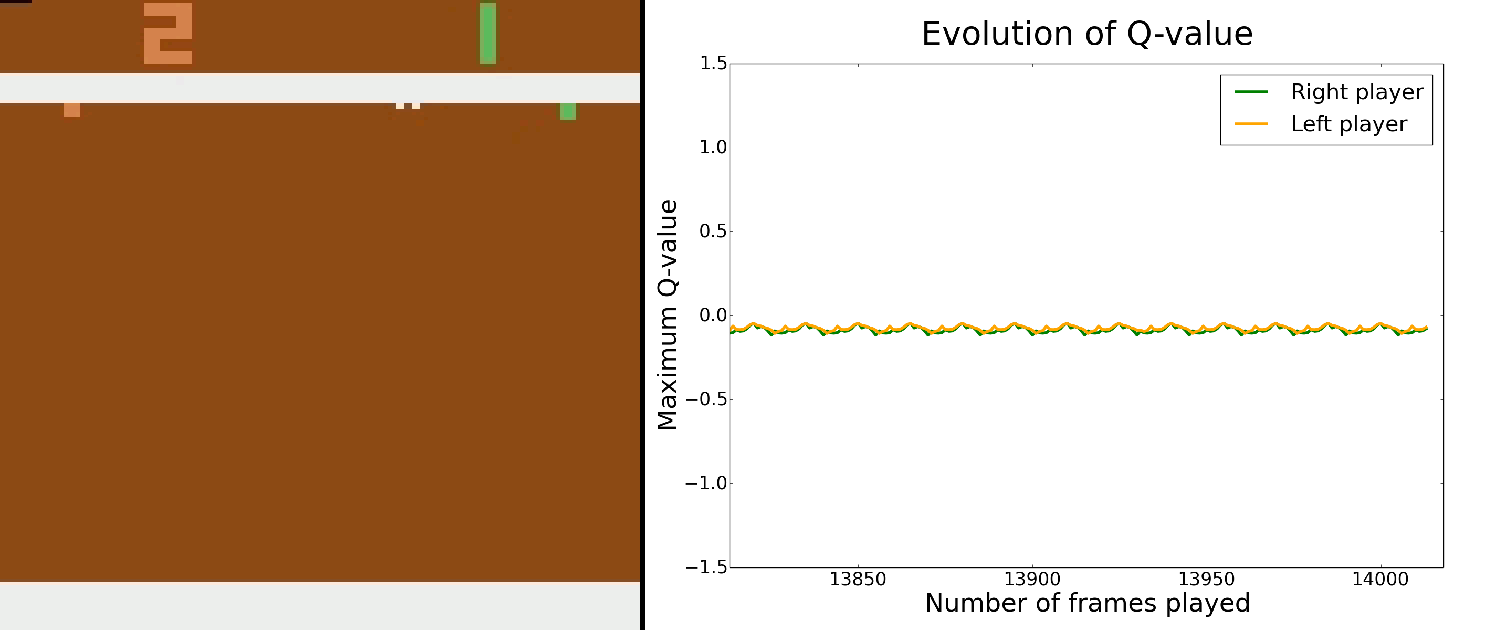

Supplement: S2 Fig — By placing themselves at the upper border of the field and bouncing the ball between themselves, the cooperative agents manage to keep the ball in the game indefinitely. (TIF) [file pone.0172395.s002.tif]
