# Supplementary material for: Multiagent cooperation and competition with deep reinforcement learning
Source: PLoS One. 2017 Apr 5;12(4):e0172395. doi: 10.1371/journal.pone.0172395 (PMC5381785; doi:10.1371/journal.pone.0172395)
Supplement: S1 Text — (PDF) [file pone.0172395.s003.pdf]

Adaptation of the Code for the Multiplayer Paradigm

The original code published with [1] does not provide the possibility to play multiplayer games. Whereas the agents are independent realizations of DQNs and many of the Atari games allow multiple players, the communication protocol between the agents and the emulator restricts one to use a single-player. To solve this issue we had to modify the code to allow transmitting actions from two agents, as well as receiving two sets of rewards from the environment. The game screen is fully observable and is shared between the two agents, hence no further modifications were needed to provide the state of the game to multiple agents simultaneously.

References

1. Mnih V, Kavukcuoglu K, Silver D, Rusu AA, Veness J, Bellemare MG, et al. Human-level control through deep reinforcement learning. Nature. 2015;518(7540):529–533.
