# Supplementary material for: Multiagent cooperation and competition with deep reinforcement learning
Source: PLoS One. 2017 Apr 5;12(4):e0172395. doi: 10.1371/journal.pone.0172395 (PMC5381785; doi:10.1371/journal.pone.0172395)
Supplement: S2 Text — (PDF) [file pone.0172395.s004.pdf]

Game selection

Atari Learning Environment (ALE) [1] currently supports 61 games, but only a handful on them have a two player mode. In order to choose a suitable game for our multiplayer experiments we used following criteria:

1. The game must have real-time two-player mode. Many games (e.g. *Breakout*) alternate between two players and are therefore less suitable for our multiagent learning experiments.
2. Deep Q-learning algorithm must be able to play the game above human level in single-player mode. For example *Wizard of Wor* has a two-player mode, but requires extensive labyrinth navigation, which current deep Q-learning algorithm is not able to master.
3. The game must have a natural competitive mode. In addition, we were interested in games, where we can switch between cooperation and competition by a simple change of reward function.

After having considered several other options we finally chose to work within the *Pong* game environment because it satisfies all the criteria, it was supported by existing code and can be learned relatively quickly. It also has the advantage of being easily understood by the reader due to its simplicity and its role in the video game history.

While this is outside the scope of the present work, we would like to report on two other games which might mix well with interesting scientific questions on competition and collaboration. Firstly, *Outlaw* is a simple shooting game that in a restricted game mode can be seen as a real-time approximation of prisoner's dilemma. Secondly, *Warlords* is a game with up to four players where the emergence of collaboration in the face of an adversary could be tested.

References

1. Bellemare MG, Naddaf Y, Veness J, Bowling M. The arcade learning environment: An evaluation platform for general agents. arXiv preprint arXiv:12074708. 2012;.
