# Supplementary material for: Multiagent cooperation and competition with deep reinforcement learning
Source: PLoS One. 2017 Apr 5;12(4):e0172395. doi: 10.1371/journal.pone.0172395 (PMC5381785; doi:10.1371/journal.pone.0172395)
Supplement: S3 Text — (PDF) [file pone.0172395.s005.pdf]

Comparison of single-player and multiplayer training

To understand the strategy of the single-player DQN, we need to first describe its opponent - the Pong algorithm. The built-in Pong algorithm follows a simple hard-wired strategy - it tries to keep the upper-central part of the paddle at the same height as the ball at all times. The algorithm is deterministic and no random actions are introduced. Moreover, when the ball is served, the paddle controlled by Pong algorithm is reset to be at the center of the field. As a result, the Pong controller never makes mistakes with most types of shots, but is limited to always react the same way to the same trajectories. As the speed of movement of the Pong agent is limited (slower than that of the DQN agent's), it is impossible to follow the height of balls flying with a high vertical velocity. This is the one flaw in the algorithm.

Single-player DQN has been trained by making it play against the Pong algorithm. It has figured out that balls flying with high vertical velocity are the only ones that can beat the opponent and therefore it makes the ball fly at an acute angle whenever it can. Very often we can see a stereotypical behaviour i) ball is served towards the Pong agent and it responds in a deterministic manner leading to the ball arriving to a specific spot on the other side, ii) the single-player DQN is waiting for the ball and hits it with the side of the paddle, giving it a high vertical velocity and making it impossible for the hard-coded algorithm to catch it. The sinlge-player DQN wins matches against the Pong algorithm with an average score of 21-3, which is a very good result.

The strategy of hitting balls at acute angles does not always work against other opponents. The newly introduced hand-coded agents can move the paddle faster than the Pong algorithm and can reach some of the balls with high vertical velocity. Multiagent DQN can move faster and can also plan ahead and position itself wherever it wants, instead of tracking the ball. This means that scoring points is more difficult for the single-player DQN against these new opponents.

As an additional difficulty, the single-player agent is accustomed to the opponent always moving the same way. Pong algorithm reacts in a deterministic manner to stereotypical game situations such as when the ball is served. As the opponent is part of the visual input that the DQN receives, a new opponent choosing a different position on the field leads to a different input. Such unusual inputs lead single-player DQN to make mistakes that it never makes when playing against the Pong algorithm. The observed mistakes are, for example, failing to catch a ball served towards it and not reacting at all even to relatively slow balls. As a result, despite being dominant against the Pong algorithm, the single-player DQN loses against all the new opponents, including the multiplayer DQN.

Multiplayer DQNs are free of both types of biases - they have played against an opponent capable of learning to deal with any type of shots. Also their opponent, another DQN, moves around the field freely, not limited to tracking the height of the ball. Also, in multiplayer game agent positions are not reset when the ball is served. As a result multiplayer agents receive more diverse inputs when training, leading to more robust strategies. This is demonstrated by an increased performance against the new hand-coded algorithms as well as against the single-player DQN.
