# Supplementary material for: Multiagent cooperation and competition with deep reinforcement learning
Source: PLoS One. 2017 Apr 5;12(4):e0172395. doi: 10.1371/journal.pone.0172395 (PMC5381785; doi:10.1371/journal.pone.0172395)
Supplement: S4 Text — (PDF) [file pone.0172395.s006.pdf]

Access to code

The version of the code adapted to the multiplayer paradigm together with the tools for the visualization can be accessed at our Github repository:
https://github.com/NeuroCSUT/DeepMind-Atari-Deep-Q-Learner-2Player.
