# Supplementary material for: Multiagent cooperation and competition with deep reinforcement learning
Source: PLoS One. 2017 Apr 5;12(4):e0172395. doi: 10.1371/journal.pone.0172395 (PMC5381785; doi:10.1371/journal.pone.0172395)
Supplement: S5 Text — (PDF) [file pone.0172395.s007.pdf]

Access to videos

Please see the videos illustrating the core concepts and the results of the current work on the dedicated YouTube playlist `https://www.youtube.com/playlist?list=PLfLv_F3r0TwyaZPe5OOOUx8tRf0HwdR_u`.
