# Supplementary material for: Multiagent cooperation and competition with deep reinforcement learning
Source: PLoS One. 2017 Apr 5;12(4):e0172395. doi: 10.1371/journal.pone.0172395 (PMC5381785; doi:10.1371/journal.pone.0172395)
Supplement: S1 Table — Rewarding schemes used to explore the behaviours between competitive and the cooperative strategy. Rewards in columns 2 and 3 must be multiplied with the normalization coefficient given in the 4th column to make the learning signal equally strong in all rewarding schemes. (PDF) [file pone.0172395.s008.pdf]

Normalization of rewarding schemes

Rewarding scheme	Unnormalized reward for scoring	Unnormalized reward for conceding	Normalization coefficient
Fully competitive, $\rho = 1$	1	-1	1
$\rho = 0.5$	0.5	-1	1.333(3)
$\rho = 0.25$	0.25	-1	1.6
$\rho = 0$	0	-1	2
$\rho = -0.25$	-0.25	-1	1.6
$\rho = -0.5$	-0.5	-1	1.333(3)
Fully cooperative, $\rho = -1$	-1	-1	1

Table 1. Rewarding schemes to explore the behaviours between competitive and the cooperative strategy. Rewards in columns 2 and 3 must be multiplied with the normalization coefficient given in the 4th column to make the learning signal equally strong in all rewarding schemes.
