# Supplementary material for: Multiagent cooperation and competition with deep reinforcement learning
Source: PLoS One. 2017 Apr 5;12(4):e0172395. doi: 10.1371/journal.pone.0172395 (PMC5381785; doi:10.1371/journal.pone.0172395)
Supplement: S2 Table — Behavioural statistics of the agents as a function of their incentive to score. (PDF) [file pone.0172395.s009.pdf]

Table of behavioral measures for ”Progression from competition to collaboration”

Agent	Average paddle-bounces per point	Average wall-bounces per paddle-bounce	Average serving time per point
Competitive $\rho = 1$	7.21 ± 1.03	0.85 ± 0.08	113.29 ± 39.47
Transition $\rho = 0.75$	6.74 ± 0.59	0.91 ± 0.07	146.93 ± 24.33
Transition $\rho = 0.5$	6.76 ± 0.44	0.82 ± 0.07	323.13 ± 60.89
Transition $\rho = 0.25$	7.06 ± 0.79	0.71 ± 0.05	286.32 ± 74.24
Transition $\rho = 0$	7.06 ± 1.00	0.50 ± 0.05	411.14 ± 85.89
Transition $\rho = -0.25$	8.44 ± 0.39	0.42 ± 0.05	446.57 ± 109.23
Transition $\rho = -0.5$	8.62 ± 1.31	0.51 ± 0.08	437.70 ± 109.98
Transition $\rho = -0.75$	67.29 ± 103.30	0.05 ± 0.13	378.02 ± 75.42
Cooperative $\rho = -1$	421.25 ± 341.44	0.01 ± 0.00	416.80 ± 136.06

Table 1. Behavioural statistics of the agents as a function of their incentive to score.
